# A rapid review to inform the policy and practice for the implementation of chronic disease prevention and management programs for Aboriginal and Torres Strait Islander people in primary care

**DOI:** 10.1186/s12961-024-01121-x

**Published:** 2024-03-21

**Authors:** Uday Narayan Yadav, Jasmine Meredith Davis, Keziah Bennett-Brook, Julieann Coombes, Rosemary Wyber, Odette Pearson

**Affiliations:** 1 National Centre for Aboriginal and Torres Strait Islander Wellbeing Research, Australian National University, Canberra, ACT Australia; 2 Centre for Primary Health Care and Equity, University of New South Wales, Sydney, Australia; 3 Melbourne Medical School, The University of Melbourne, Melbourne, Australia; 4 The George Institute for Global Health, Sydney, Australia; 5 Telethon Kids Institute, Perth, WA Australia; 6 South Australian Health and Medical Research Institute, Adelaide, SA Australia; 7 Faculty of Health and Medical Science, University of Adelaide, Adelaide, SA Australia

**Keywords:** Aboriginal and Torres Strait Islander people, Barriers and enablers Chronic disease prevention and management, Primary health care

## Abstract

**Background:**

More than 35% of Aboriginal and Torres Strait Islander adults live with cardiovascular disease, diabetes, or chronic kidney disease. There is a pressing need for chronic disease prevention and management among Aboriginal and Torres Strait Islander people in Australia. Therefore, this review aimed to synthesise a decade of contemporary evidence to understand the barriers and enablers of chronic disease prevention and management for Aboriginal and Torres Strait Islander People with a view to developing policy and practice recommendations.

**Methods:**

We systematically searched for peer-reviewed published articles between January 2014 to March 2023 where the search was performed using subject headings and keywords related to “Aboriginal and Torres Strait Islander peoples,” “Chronic Disease,” and “Primary Health Care”. Quality assessment for all included studies was conducted using the Aboriginal and Torres Strait Islander Quality Appraisal Tool. The data were extracted and summarised using a conventional content analysis approach and applying strength-based approaches.

**Results:**

Database searches identified 1653 articles where 26 met inclusion criteria. Studies varied in quality, primarily reporting on 14 criteria of the Aboriginal and Torres Strait Islander Quality Appraisal Tool. We identified six key domains of enablers and barriers of chronic disease prevention and management programs and implied a range of policy and practice options for improvement. These include culturally acceptable and safe services, patient-provider partnerships, chronic disease workforce, primary health care service attributes, clinical care pathways, and accessibility to primary health care services. This review also identified the need to address social and cultural determinants of health, develop the Aboriginal and Torres Strait Islander and non-Indigenous chronic disease workforce, support multidisciplinary teams through strengthening clinical care pathways, and engage Aboriginal and Torres Strait Islander communities in chronic disease prevention and management program design and delivery.

**Conclusion:**

Enabling place-based partnerships to develop contextual evidence-guided strategies that align with community priorities and aspirations, with the provision of funding mechanisms and models of care through policy and practice reforms will strengthen the chronic disease prevention and management program for Aboriginal and Torres Strait Islander people.

**Supplementary Information:**

The online version contains supplementary material available at 10.1186/s12961-024-01121-x.

## Introduction

Aboriginal and Torres Strait Islander people have continuously demonstrated strength, tenacity, and resilience in the face of a high burden of chronic disease associated with profound health, social, economic and cultural and wellbeing impacts. The disproportionate burden of chronic disease—particularly cardiovascular disease (CVD), type II diabetes and chronic kidney disease (CKD)—is driven by the effects of colonisation. These effects include intergenerational trauma, racism and commercial determinants such as the introduction of ultra-processed foods, tobacco, sugar-sweetened beverages, and alcohol [1, 2].

More than 35% of Aboriginal and Torres Strait Islander adults report having CVD, diabetes or CKD, 38% have two of these conditions and 11% have all three [3]. Nearly 70% of Australia’s burden of disease from CVD is attributable to modifiable risk factors including high blood pressure, dietary risks, high body weight, high cholesterol and smoking [4]. These risk factors, and subsequent disease, can be prevented. This prevention can be primordial (by addressing the structural drivers at a population level, such as food supply and recreation facilities) or primary (through identifying people at risk and taking individual steps to reduce that risk).

Primary prevention and management of chronic disease among Aboriginal and Torres Strait Islander people in Australia predominantly occurs in primary care settings. Universal primary care in Australia is largely funded through a fee-for-service model via the Medicare Benefits Scheme (MBS), with some augmentation for Aboriginal and Torres Strait Islander people through the Indigenous Australian’ Health Programme [5]. Primary care is delivered by a range of providers, including Aboriginal Community Controlled Health Organisations (ACCHOs) which are governed by a local board, Aboriginal Medical Services run by State and Territory governments, and private primary care services (sometimes referred to as ‘mainstream’ providers). Chronic disease services in primary care include risk assessment, support for healthy behaviours, referrals to allied health and pharmacology for risk reduction. High quality, culturally safe primary care can prevent the development of disease, and manage complications, for individuals and communities [6–8]. Strengthening primary care is a key strategic priority of the Australian government, [9, 10] peak bodies and other stakeholders working to improve the health and well-being of Aboriginal and Torres Strait Islander communities [11, 12].

Understanding how primary care services, prevention and management programs can best meet the needs of Aboriginal and Torres Strait Islander people, who are living with or at risk of chronic disease, is critical to addressing disparity in outcomes. A review conducted by Gibson et al. in 2015 explored chronic disease care for Indigenous communities globally, but there has been no comprehensive contextual evidence available focusing on Aboriginal and Torres Strait Islander people [13]. Therefore, there is a need for local evidence disease on chronic prevention and management for Aboriginal and Torres Strait Islander people in Australia. The Aboriginal and Torres Strait Islander People’s Health Assessment (MBS item number 715) is an annual health check funded for Aboriginal and Torres Strait Islander people and a cornerstone of early detection for chronic disease [14]. The number of Health Assessments fell for the first time in 2020 and 2021 after years of sustained growth [15]. The reduction in Health Assessments is likely to reflect disruption to routine primary care services during the COVID-19 pandemic [16].

Our team were contracted by the Australian Commonwealth Department of Health and Aged Care to understand best practice delivery of chronic disease care for Aboriginal and Torres Strait Islander people in 2020. The effects of the COVID-19 pandemic accelerated the need for this research and necessitated a pivot to rapid review strategy. The Oxford Centre for Evidence-Based Medicine highlights [17] that rapid review methodology can be used to meet the needs of commissioning bodies in policy-relevant timeframes. Therefore, this rapid review aims to synthesise contemporary and contextually relevant barriers and enablers for chronic disease care in primary care settings for Aboriginal and Torres Strait Islander people with a view to developing near term policy and practice recommendations.

## Methods

This review applied the SelecTing Approaches for Rapid Reviews (STARR) decision tool that includes interaction with commissioners, scoping the literature, selecting approaches to literature search, methods for data extraction and evidence synthesis [18]. It was conducted based on the Preferred Reporting Items for Systematic reviews and Meta-Analyses extension for Scoping Reviews (PRISMA-ScR) guideline [19]. We searched two databases (Medline and Web of Science) to identify relevant studies from January 2014 to March 2023. These two databases were chosen because of following reasons: (i) Medline database provides access to articles from 5,200 journals in about 40 languages covering the biomedical and life sciences including Aboriginal and Torres Strait Islander peoples as a MeSH Headings and (ii) Web of Science is the world’s oldest interdisciplinary and widely used database of research publications that covers over 34,000 journals today.

This timeframe follows on from a systematic review conducted by Gibson et al. [13] which included data up to December 2013. This rapid review mirrors the Gibson methodology with two main changes: i) narrowing focus from Indigenous communities globally to Aboriginal and Torres Strait Islander contexts in Australia, ii) narrowing focus from all chronic diseases to focus on the three major contributors to chronic disease burden (cardiovascular disease, diabetes and chronic kidney disease). The review was performed between January to August 2023.

In this review, Aboriginal and Torres Strait Islander researchers and *Thiitu Tharrmay Aboriginal and Torres Strait Islander Reference Group* members were actively engaged and consulted in all steps, from the inception of the research questions to the completion of this review. Throughout the process, the research team met monthly with the Australian Commonwealth Department of Health and Aged Care during the period of this review to inform the scope of evidence synthesis.

**Positionality:** The senior author of this review Pearson (nee Gibson, first author of the 2015 review), is *Kuku Yalnji/Torres Strait Islander* health systems researcher. KB-B (*Torres Strait Islander*) and JC (*Gumbaynggir*) are experienced researchers with particular expertise in quality assessment of Aboriginal and Torres Strait Islander research studies. UNY (*Madhesi, Nepal*) is an implementation scientist, JD is a non-Indigenous medical student and RW is a non-Indigenous practising general practitioner and researcher.

### Operational definition of key terminology used in this review:

- Holistic care or support: The care process that involved strategies to support mental, physical, cultural and spiritual health which is beyond the individual level that values family and community capacity and governance [20].
- Systems thinking: Systems thinking is c*onceptual* orientation concerned with inter-relationships between different levels, institutions, systems, and people nested within social, cultural, economic, political contexts to deliver a holistic care [21].
- Place-based partnerships: Place-based partnerships involve collaborative arrangements the unique needs and circumstances of both the community and service provider. In this context, place-based partnerships involve a formal partnership among government, service providers and First Nations representatives. These partnerships are specific to geographical locations and population groups and are aimed at designing or delivering services that directly respond to community needs, aspirations and local priorities, while valuing local cultural values [22, 23].

### Scoping the literature:

**Fig. 1:**
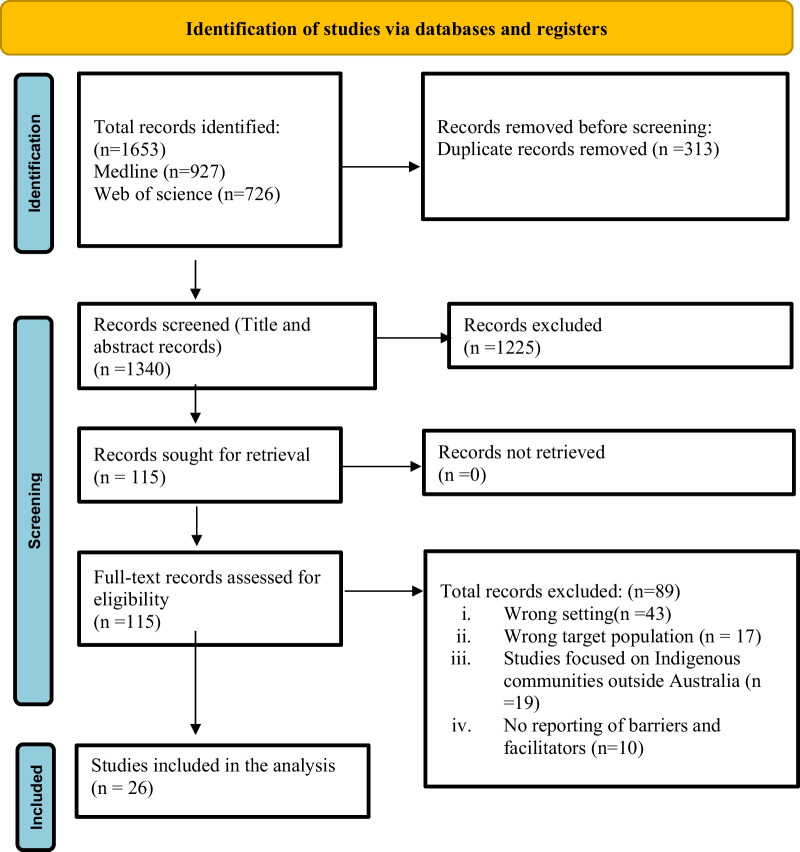
PRISMA 2020 flow diagram that included searches of databases and included studies

The search was performed using a combination of subject headings and keywords related to “Aboriginal and Torres Islander peoples,” “Chronic Disease,” and “Primary Health Care” using “OR” and “AND” iterated from the search strategy described in a study by Gibson et al. [13] The search strategy has been provided in Box 1. The search results obtained from two databases were imported to Endnote and uploaded on the Covidence platform for title and abstract and full-text screening [24]. Three reviewers (UNY, JMD and RW) independently screened the titles and abstracts of potential studies for eligibility based on inclusion and exclusion criteria (Table 1). Full-text articles were assessed by two reviewers and any disagreement that appeared during the screening process was resolved through discussion with the third reviewer (RW). The details of the screening process are documented as a PRISMA flow diagram (Fig. 1).

**Table 1:** Inclusion and exclusion criteria

Inclusion criteria	✔ Aboriginal and Torres Strait Islander people of any age with a chronic disease, their family or community members, PHC providers (doctors, nurses, administrators, Aboriginal and Torres Strait Islander Health Practitioners and workers), and decision-makers working in Indigenous health in Australia ✔ Studies focus on the prevention, diagnosis, and management of chronic disease (CVD (including ischaemic heart disease, stroke, and atrial fibrillation), type II diabetes, chronic kidney disease, chronic respiratory disease) and risk factors ✔ Initiatives implemented in primary health care settings in Australia (including, outreach services) ✔ Qualitative studies, program evaluations, implementation research including RCTs, and quasi-experimental studies will be included ✔ Qualitative inquiry, field reflection notes, and descriptive surveys focusing on chronic disease care at the PHC level will be included
Exclusion	✔ Studies that are focused on mental illnesses and chronic infectious/post-infectious conditions (HIV, RHD, chronic otitis media) ✔ Guideline and policy documents ✔ Editorials, commentaries, and systematic reviews ✔ Studies focused on Indigenous communities outside Australia ✔ Studies conducted outside PHC setting (i.e., occupational screening programs) ✔ Quantitative studies investigated the effectiveness of CD interventions by measuring health outcomes rather than factors enabling or inhibiting their implementation ✔ No segregated data for Indigenous Australians in an Australian or multicounty research that included both Indigenous and non-indigenous data but did not have specific indigenous data analysis ✔ Prevalence studies that do not report factors enabling or inhibiting their implementation will be excluded

## Box 1: Search strategy

**Table:** 

1. PubMed/Medline	
Search#1	(Indigenous [tiab] OR Aborigin* [tiab] OR Torres Strait Islander [tiab] OR First Nation [tiab] OR Oceanic Ancestry Group [Mesh])
Search#2	(Chronic disease [tiab] OR Chronic illness [tiab] OR Cardiovascular disease [tiab] OR Heart disease [tiab] OR Atherosclerosis [tiab] OR Stroke [tiab] OR Arrhythmia [tiab] OR Heart attack [tiab] OR Myocardial infarction [tiab] OR Hypertension [tiab] OR Kidney disease [tiab] OR renal disease [tiab] OR Diabet* [tiab] OR Chronic disease [Mesh] OR Cardiovascular disease [Mesh] OR Kidney Diseases [Mesh] OR Diabetes Mellitus [Mesh])
Search#3	(Primary health [tiab] OR primary care [tiab] Community [tiab] OR rural [tiab] OR Remote [tiab] OR Outreach [tiab] OR intervention [tiab] OR program* [tiab] OR general practice [tiab] OR "Primary health care" [Mesh] OR "Health Services, Indigenous" [Mesh])
Search#4	Australia
Search#5	#1 AND #2 AND #3 AND #4
Limits: Publication date from 01/01/2014 to 28/03/2023; English language	
**2. Web of Science**	
(((ALL = (Chronic disease OR Chronic illness OR Chronic respiratory disease OR Obstructive lung disease OR Chronic obstructive pulmonary disease OR Bronchiectasis OR Asthma OR Cardiovascular disease OR Heart disease OR Atherosclerosis OR Stroke OR Arrhythmia OR Heart attack OR Myocar Results for (((ALL = (Chronic disease OR Chronic illness OR Chronic respiratory disease OR Obstructive lung disease OR Chronic obstructive pulmonary disease OR Bronchiectasis OR Asthma OR Cardiovascular disease OR Heart disease OR Atherosclerosis OR Stroke OR Arrhythmia OR Heart attack OR Myocardial infarction OR Hypertension OR Kidney disease OR renal disease OR Diabetes Or depression OR Chronic disease OR Respiratory Tract Diseases OR Cardiovascular disease OR Kidney Diseases OR Diabetes Mellitus)) AND ALL = (Primary health OR primary care OR intervention OR program OR general practice OR Primary health care OR Health Services)) AND ALL = (Indigenous OR Aboriginal OR Torres Strait Islander OR First Nation)) AND ALL = (Australia) and Review Article (Exclude—Document Types) and Letter or Meeting Abstract or Editorial Material (Exclude—Document Types) and Proceeding Paper or Early Access or Data Paper or Correction (Exclude—Document Types)dial infarction OR	

### Data extraction

**Fig. 2:**
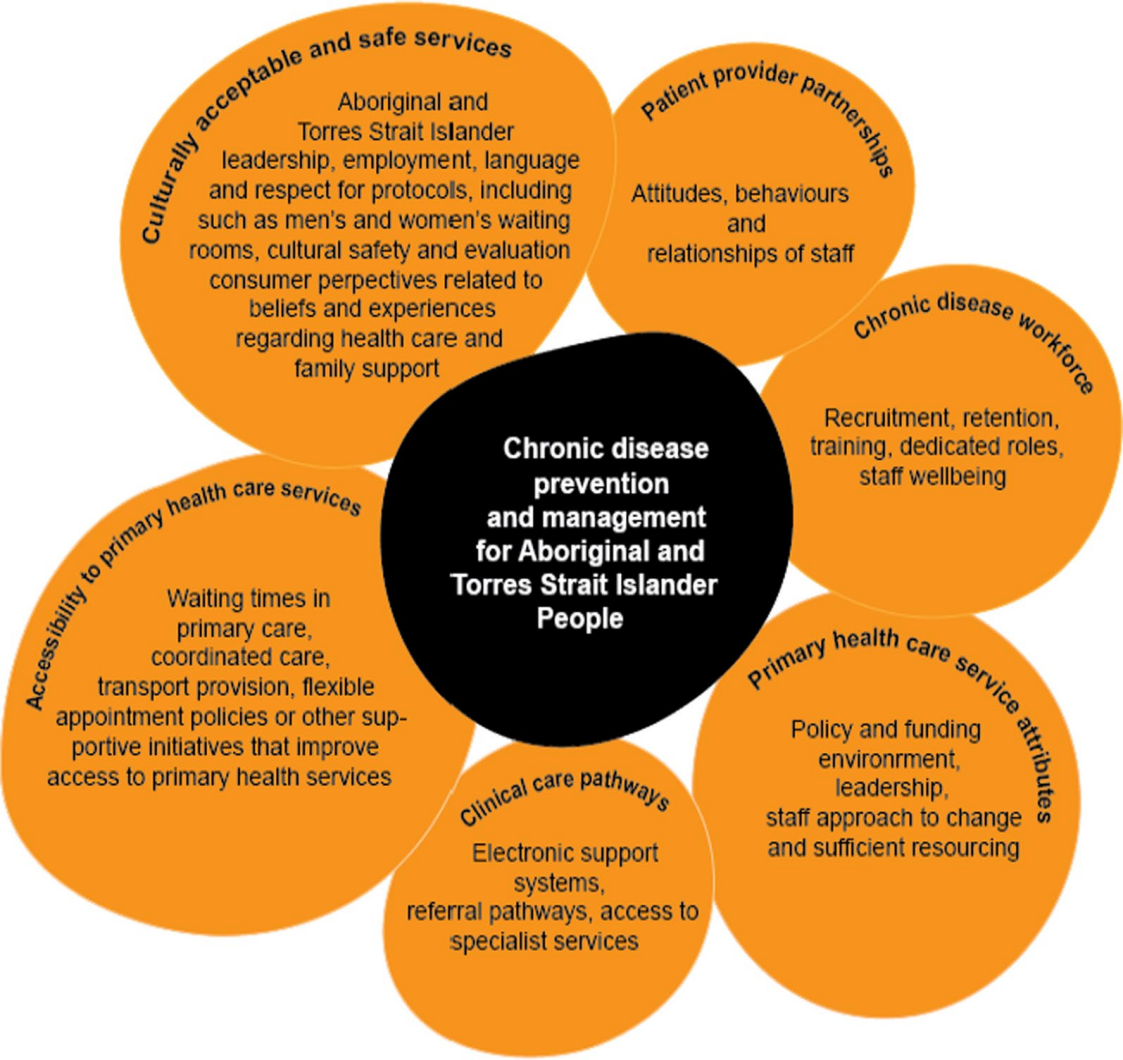
Barriers and enablers to chronic disease prevention and management for Aboriginal and Torres Strait Islander people

An iterative process was used to define data extraction domains. A total of fourteen potential domains were identified from the systematic review conducted by Gibson et al., alongside the *He Pikinga Waiora* Implementation Framework [25] and an access framework for Aboriginal and Torres Strait Islander people [26]. Further, three discussions were held within the team and with other Aboriginal and Torres Strait Islander researchers that identified eight domains of interest for both barriers and enablers (design attributes, chronic disease workforce, patient/provider partnership, clinical care pathways, access-accessibility, access-acceptability, system thinking and knowledge translation). A data extraction tool was prepared to extract information about the study characteristics (title, author, publication year, study setting, study objective, study design, types of services) and eight domains decided from the discussions mentioned above. A data extraction tool was shared with the *Thiitu Tharrmay Aboriginal and Torres Strait Islander Reference Group* members at the National Centre for Aboriginal and Torres Strait Islander Wellbeing Research at the Australian National University for their input. Based on inputs from *Thiitu Tharrmay Aboriginal and Torres Strait Islander Reference Group* members and team discussion, some domains were consolidated and made an agreement of extracting data focusing six domains (culturally acceptable and safe services, patient provider partnerships, chronic disease workforce, primary health care service attributes, clinical care pathways and accessibility to primary health care services) the final data extraction tool included study characteristics and six domains (Fig. 2)focusing both barriers and enablers. The data extraction tool was piloted on five included studies that facilitated shared understanding of approach to data extraction between team members. The data were extracted by UNY and JMD between May to June 2023.

### Evidence synthesis and quality appraisal

Quality assessment for all included studies was conducted using the Aboriginal and Torres Strait Islander Quality Appraisal Tool that privileges Aboriginal and Torres Strait Islander people’s (knowing) epistemology, (being) ontology, (doing) axiology and ethical research governance [27]. The extracted data were analysed and summarised using a conventional content analysis approach [28] that allows the categories and the name of the categories to flow from the data and applying strength-based approaches [29].

## Findings

### Study selection and its characteristics

For the period 2013- 2023, a total of 1653 articles were retrieved from Medline (*n* = 927) and Web of Science (*n* = 726) databases. Of the total, 313 were duplicates which left 1340 articles for screening. Following the screening, 115 studies were selected for the full-text review. Upon full-text review, 89 articles were excluded, leaving 26 articles to be included for extraction and evidence synthesis. Service delivery models of the included studies were ACCHOs, government-run Aboriginal Medical Services and private general practice. The majority of the studies applied qualitative or mixed-method evaluation approaches (see Additional file 1).

### Quality appraisal results

All studies met two criteria from the Aboriginal and Torres Strait Islander Quality Appraisal Tool: priority determined by community (criterion 1) and use of an Indigenous research paradigm (criterion 9). Over 92% studies met criteria for community protocols (criterion 5), 80% of studies met criteria addressing community consultation and engagement (criterion 2), Aboriginal and Torres Strait Islander leadership (criterion 3) and 76% studies had demonstrated Aboriginal and Torres Strait Islander governance in research (criterion 4). While 30% or less of the studies addressed existing intellectual and cultural property (criterion 6 and 7), 70% studies met the following criteria: Aboriginal and Torres Strait Islander people control over collection and management of research materials (criteria 8), use of strength based approach and acknowledging practices that have harmed Aboriginal and Torres Strait Islander communities (criterion 10) translation of findings into sustainable changes (criterion 11), benefit the participants and Aboriginal and Torres Strait Islander communities (criterion 12), demonstrate capacity strengthening for Aboriginal and Torres Strait Islander people (criterion 13), and researchers have opportunities to learn from each other (criterion 14). Details are provided in Table 2.

**Table 2:** Quality appraisal of the included studies

s.no.	Authors	Question number of Aboriginal and Torres Strait Islander Quality Appraisal Tool														
		1	2	3	4	5	6	7	8	9	10	11	12	13	14	Total
1	Cuesta-Briand 2014	1	1	0	1	1	0	0	1	1	1	0	0	0	0	7
2	Deshmukh 2014	1	1	0	1	1	0	0	0	1	1	0	1	0	0	7
3	Govil 2014	1	1	0	1	1	0	0	1	1	1	0	1	0	1	9
4	Stoneman 2014	1	0	1	0	1	0	0	0	1	1	0	0	0	0	5
5	Bailie 2015	1	1	1	0	1	0	0	0	1	1	1	1	0	1	9
6	Barrett 2015	1	1	1	1	1	0	0	1	1	1	1	1	1	1	12
7	Reev 2015	1	1	1	1	1	0	1	1	1	1	1	1	1	1	13
8	Schmidt 2015	1	0	0	0	1	0	0	0	1	1	0	0	0	1	5
9	Bailie 2016	1	1	1	1	1	0	0	1	1	1	1	1	1	1	12
10	Askew 2016	1	1	1	1	1	0	0	1	1	1	1	1	1	1	12
11	Conway 2017	1	1	1	0	1	0	0	1	1	1	1	1	1	1	11
12	Bailie 2017	1	1	1	1	1	0	1	1	1	1	1	1	1	1	13
13	Cambell 2017	1	0	1	1	1	0	0	1	1	0	1	1	1	1	10
14	Kirkham 2017	1	1	1	1	1	1	0	1	1	0	1	1	1	1	12
15	Davy 2017	1	1	1	1	1	1	1	1	1	1	1	1	1	1	14
16	Spurling 2017	1	1	1	0	0	1	0	1	1	1	1	1	1	1	11
17	Webster 2017	1	1	0	0	1	0	0	0	1	1	0	0	1	1	7
18	Wood 2017	1	0	1	1	0	1	0	1	1	1	1	1	1	1	11
19	Canuto 2018	1	1	1	1	1	1	0	1	1	1	1	1	1	1	13
20	Kirkham 2019	1	1	1	1	1	1	0	1	1	0	1	1	1	1	12
21	Macniven 2019	1	1	1	1	1	0	0	0	1	0	1	0	1	0	8
22	Seear 2019	1	1	1	1	1	0	0	1	1	1	1	1	1	1	12
23	Sebastian 2020	1	1	1	1	1	0	0	1	1	0	1	1	1	1	11
24	Seear 2020	1	1	1	1	1	1	0	1	1	1	1	1	1	1	13
25	Wood 2021	1	1	1	0	1	0	0	1	1	1	1	1	1	1	11
26	Blignault 2021	1	1	1	1	1	0	1	1	1	1	1	1	1	1	13

Answer **1** for yes or partially for each question, Answer **0** for no or unclear for each question

### Enablers and barriers to the implementation of chronic disease initiatives

Enablers and barriers (see Additional file 2) are presented under six thematic domains and relevant sub-themes presented below:

- Primary health care service attributes
- Aboriginal and Torres Strait Islander engagement and Aboriginal leadership in system design: Aboriginal and Torres Strait Islander project leadership and community engagement were overwhelmingly identified as key determinants for system design [30–47]. Engagement through outreach cultural community events and stakeholder partnerships appear to amplify these effects [48]. Six studies investigated how information was exchanged between knowledge-users and researchers throughout the studies [30, 38, 44–47]. Knowledge users included Aboriginal and Torres Strait Islander communities, primary health care providers (ACCHOs and mainstream providers), Aboriginal and/ or Torres Strait Islander health workers and practitioners, other service providers, and policymakers. Alongside this, one study acknowledged the utilisation of cultural and scientific evidence to provide best-practice healthcare [37]. Aboriginal and Torres Strait Islander project leadership consistently facilitated trust and satisfaction. Use of evidence-based clinical care guideline in project implementation within community and services were other key benefits of knowledge translation.
- Primary health car responsive to local community needs: Prospective analysis of potential barriers for program development were essential to program success, in order to actively address these in the program design and implementation. Many studies clearly demonstrated how barriers were overcome through tailored solutions including: outreach services to save patient time and cost [30, 31, 45, 49]; free of charge services [31, 49–51]; 24-h culturally safe service [32, 37, 50]; and telehealth specialist services [51, 52]. Outreach services (delivered outside of the clinic facility) included home medication delivery, general visits by health workers to build trust and screening for conditions at community-based events. Services which were free at point of care included medications, nicotine patches, hospital specialist clinics, supplementary services (e.g. cooking class, aged care services), and preventive initiatives (e.g. blood pressure measurement, glucose monitor and health promotion activities). Innovative primary care models for Aboriginal and Torres Strait Islander patients included flexible appointment systems [32, 37, 50], multidisciplinary teams for providing holistic care [37, 38, 51], and clinical audits for quality improvement were also effective [31, 34, 39, 45, 46]. Incorporating chronic disease prevention into primary care occurred through health promotion initiatives [30, 35, 37, 50, 51], and patient-led prevention initiatives and management plans [30, 46, 50] were also found to be invaluable in addressing community needs. Six studies explicitly emphasised the importance of adequate resources and flexible funding for primary care services to meet local Aboriginal and Torres Strait Islander community priorities and needs [30, 32, 36–39].
- A holistic approach to care: Aligned with Aboriginal and Torres Strait Islander peoples’ perception of health**,** holistic support [30, 38, 44] and care coordination was critical, necessitating flexible funding, and systems thinking and innovative, locally-adapted reforms [30, 31, 46]. Social referral approaches that connected people to non-clinical services were an important component in Aboriginal community controlled primary care settings, for example: connecting patients to exercise groups, providing access to housing, opportunities for hobbies, or home care services such as ‘Meals on Wheels’ [38, 40, 41, 53].
- Primary care access: Several barriers were identified, including: a lack of support specific to Aboriginal and Torres Strait Islander leadership [34, 39, 51, 53, 54]; competing priorities of healthcare service delivery [30, 34, 39]; and a lack of funding specifically address social determinants of health [31, 55]. These were more pronounced in Aboriginal PHC. There were also insufficient resources to engage stakeholders in the co-development of primary care programs. This contributed to poor connections and relationships between multidisciplinary teams in health centres and other actors, including clinical information management systems [34, 48, 51, 54], and high turnover of trained staff [34, 50, 54]. Some studies highlighted challenges in creating culturally appropriate services, due to the heterogeneity of Aboriginal and Torres Strait Islander populations [32, 39, 55]. Moreover, accessing primary care services was particularly challenging for people with the greatest health needs [31], including those with limited health literacy which made it difficult for people to engage with chronic disease care [48, 49].
- Chronic disease workforce
- Creating supportive environment and building capacity of primary healthcare workforce: Enabling safe and good work environments for Aboriginal and Torres Strait Islander Health Workers and Practitioners [30, 35, 54], and a shared sense of purpose amongst staff to address the complex needs of patients [30, 47] were consistently identified as enablers. Eight studies highlighted the importance of dedicated staff for chronic disease management, with clear delegation of responsibilities and a positive team culture created through an engaging and collaborative work environment [30, 36, 37, 39, 43, 46, 51, 54]. Establishing the health care workforce with chronic disease ‘portfolios’ were considered more able to provide recurrent, culturally safe, preventative, and responsive healthcare for Aboriginal and Torres Strait Islander people because they had a greater chance of forming trusting relationships [32, 41, 48, 50]. Included studies highlighted the need for training and development of the primary healthcare workforce [30, 31, 33, 55]. Four studies had a particular focus on local Aboriginal and Torres Strait Islander Health Workers and Practitioners including the importance of recruitment and retention strategies [31, 34, 39, 46], alongside a need to clarify their roles, provide Aboriginal and Torres Strait Islander role models [40, 41, 50, 52], and ensure they are involved in clinical decision making [30, 33, 35, 37, 39, 43, 44, 52, 54]. The importance of intensive cultural safety training for staff to deliver safe care was emphasised in five studies [37, 47, 49, 54, 55].
- Barriers to sustainable chronic disease workforce at primary health care services: Nine studies noted staff shortages and high staff turnover as adversely impacting continuity of care [32–34, 39, 43, 46, 50, 51, 53], and three studies noted inadequate clinical training for non-Indigenous staff [32, 46, 53]. Workforce limitations contributed to lack of time and resources to reach the patients that needed healthcare the most [31, 45, 46]. Nine studies specifically emphasised the substantial shortages, high turnover, and high rates of burnout of Aboriginal and Torres Strait Islander staff [33, 34, 39, 43, 46, 51, 53–55]. Work overload, inadequate support and a sense of being undervalued contributed to these issues [32, 33, 43]. Four studies noted shortages of specialists as a key barrier to integrated chronic disease management pathway [34, 43, 46, 47].
- Patient-provider partnerships
- Optimal care achieved by effective trustful patient-provider partnerships: Eight studies found that enablers of trusting patient-provider relationships included strengthening patient knowledge through interactive learning, culturally appropriate conversation, and strengths-focused clinical engagement [30, 32, 33, 35, 38, 50, 53, 55]. Numerous studies highlighted that holistic care required mechanisms for communities, families/carers and community leaders to be engaged with service providers [30, 45, 52–54]. ACCHOs were generally identified as meeting these needs by offering culturally safe care, longer consultation times to facilitate patient/provider partnerships [32, 37], and communicating with the community when there were changes in services, or the implementation of new programs [31].
- Contextual barriers to patient-provider partnership: Barriers to partnership included competing priorities for patients [53], patient experiences of racism and discrimination [32], patient discomfort with non-Indigenous services [32], and patients sensing that their holistic needs were unmet [42]. Two studies reported that limited health literacy with little shared provider-consumer understanding of chronic conditions were barriers to forming positive relationships [31, 46]. Three studies noted a general lack of connection between the clinician and patient but did not interrogate the contributors to this [32, 49, 55].
- Clinical care pathways
- Enablers to effective clinical information systems: Nine studies found in-house information technology support within primary healthcare services was crucial for effective patient referral, coordination, and follow-up care [30–32, 34, 38, 39, 43, 46, 55]. Of these, two highlighted the importance of partnership-enabled integration across health service organisations using a shared electronic health record system, disease registration multidisciplinary care plans, and a patient recall system [30, 36]. While translation of evidence-based care guidelines were not mentioned, five studies explained the importance of capacity building of staff and investment in systems development for the effective use of clinical information systems [30, 34, 39, 43, 51].
- Barriers to efficient clinical information systems: Information technology barriers were profound, including poor integration information technology systems, mixed paper and electronic records [43, 46, 51, 53], and poor infrastructure – most notably internet access [34, 52, 54]. Four studies noted shortages of trained and regular staff to implement new pathways, as a key barrier to integrated chronic disease management pathways [34, 43, 46, 47]. Inconsistent models of care [44], and poor communication between different hospital and primary care systems [50] were also barriers.
- Access to primary health care services

Many of the recognised domains of healthcare access (accessibility, accommodation, availability, accessibility and affordability) were identified [56].

- People and family-centred reforms improve access to adequate primary care: Enablers of access to primary health care services were identified in ten studies [30–32, 38–41, 43, 45, 49, 53]. Transportation support was a determining and/or motivating factor for clients to access health services [31, 38, 39] Other studies [45, 49] [43] identified accommodation factors, particularly flexible appointment systems, reduced waiting times and co-location with allied health services as key enablers. In this review, a number of motivational factors were identified for people to attend services, including support or referral from family members, higher motivation to look after oneself following the death of a family member, and motivation texts or invitational messages for health check from service providers [32, 41, 53]. In addition, one study identified providing financial incentives as an enabler for health checks [32].
- Unaddressed social determinants prevent access to primary care services: Barriers to accessing primary health care services related to socio-economic factors, health system factors and lack of health promotion factors. Socio-economic factors included accessibility and affordability considerations; lack of transport [30–32, 44, 46, 49, 50, 53–55], inability to afford health and social services, and medication costs [31, 32, 44, 47, 49]. Some studies alluded to socioeconomic factors being prioritised over primary care attendance, including household crowding and food insecurity. Ensuring that services account for competing priorities, including family and cultural responsibilities, was an important enabler [45, 55].
- System related access barriers: Health system factors included high staff turnover, lack of availability of appointments, long waiting periods, physically inaccessible clinics [43, 44, 46], poor leadership of primary care services [32, 45, 47–49], and limited internet and computer access [47]. Inadequate awareness of available services was also problematic [31, 33, 49]; initiatives run by primary care services had limited uptake when the community was not made aware of these programs [46]. Health systems need to be able to deliver services and information multimodally. For example, not all patients have phones or phone credit all the time, so several forms of communication may be required.
- Culturally acceptable and safe services
- Enablers to deliver culturally safe and acceptable services: Cultural safety is essential to the development of a mutually respectful relationship between providers and Aboriginal and Torres Strait Islander people. A systems level approach is needed to address racism experienced by Aboriginal and Torres Strait Islander people in primary care settings [30, 44]. Strategies for addressing or achieving cultural safety varied by context, but the role of Aboriginal and Torres Strait Islander healthcare workers including cultural brokerage was emphasised in two studies [31, 52]. The need for gender-specific services and gender sensitivity was emphasised by five papers as an important part of providing culturally safe care. This included delivery of programs such as gender-based exercise groups, private consultation areas for males and females, gender specific health assessment days, and employing male and female Aboriginal and Torres Strait Islander healthcare workers [31, 41, 45, 46, 52]. The provision of culturally safe services also included a need for culturally appropriate education materials, artwork, and Aboriginal and Torres Strait Islander people’s voices and images as signifiers of belonging [30, 44–46, 53]. Three studies recognised the importance of enabling Aboriginal and Torres Strait Islander patients and staff access to flexibly attend to family, community, cultural and spiritual responsibilities, and obligations to provide a culturally safe service [37, 42, 44]. One study identified the need for robust anonymous feedback systems for staff and patients to improve culturally safe care delivery [47].
- Barriers to deliver culturally safe and acceptable services: Barriers to delivering culturally safe and acceptable services related to systems, structures and lived experiences. Systems issues to providing culturally safe services included language barriers [32], poor health literacy among patients [54], long wait times due to staff shortages [32, 50, 54], a sense that services were superficial/rushed [31, 42], and lack of physical space to provide holistic care or gender-based services [43, 44, 46]. Lived experiences of treatment of Aboriginal and Torres Strait Islander people within Western systems, including health and social services, elicits feelings of harm rather than help: a fear of discrimination and racism was a key barrier to Aboriginal and Torres Strait Islander patients accessing healthcare services in five studies [31, 32, 44, 49, 53] alongside fear of diagnosis due to historical trauma [53]. These barriers were amplified where there was limited access to Indigenous-specific services [32, 44, 46]. One study mentioned a tokenistic approach where very limited community input to governance, planning, and program design was sought to develop culturally safe initiatives [46].

## Discussion

This is the first review since 2014 [13] to present the barriers and enablers of implementing chronic disease prevention and management programs for Aboriginal and Torres Strait Islander people. The enablers and barriers found in this study have several policy and practice implications that should be considered in design, implementation, and funding targets for future chronic disease prevention and management programs.

The most striking addition to our findings, relative to the 2014 [13] review, is the acknowledgment of Aboriginal and Torres Strait Islander culture (including staff, protocols, leadership, practices and ways of doing business) as a key enabler to engagement and care delivery. Partly, this is attributable to this review’s narrower focus on the Australian context. It also likely reflects increasingly detailed academic descriptions of the ways in which leadership and governance tangibly affect care delivery as part of contemporary Closing the Gap reform. All studies included in this review made some acknowledgement of culture, albeit with variations in how deeply culture was considered as an enabler of care. Our review team grappled with how best to reflect this focus on culture, given that it is both a distinct concept and intimately embedded in all thematic domains. Ultimately, we have chosen to keep Aboriginal and Torres Strait Islander culture as a separate domain to ensure that culture is given independent consideration, in addition to attention within other thematic domains. It is evident that access to culturally appropriate, affordable and comprehensive services are vital for preventing and managing chronic conditions [37]. There is no one-size-fits-all approach for models of care for Aboriginal and Torres Strait Islander communities, and programs must be tailored to local context. Recent studies have identified numerous opportunities for improving access to primary care services: creating welcoming spaces, improving the cultural safety of healthcare services, building strong trustworthy relationships between patients and providers, and building primary healthcare workforce capacity [37, 57, 58]. The span of this theme is necessarily broad, encompassing Aboriginal and Torres Strait Islander leadership, physically welcoming spaces, training for non-Indigenous staff, time to build trusting relationships. Ensuring that culture is prospectively and proactively considered in funding and delivery of primary care of chronic disease should be a priority for practitioners and policy makers.

There is clear evidence that addressing holistic needs of Aboriginal and Torres Strait Islander people enables greater engagement, rather than a narrow clinical focus on physical aspects of health. This requires primary care services to acknowledge and address the broader social and cultural determinants of health for Aboriginal and Torres Strait Islander people. Many of these have a direct impact on both chronic disease risk factors and capacity to access care (chronic disease management), including poor access to healthy and nutritious food, inadequate housing, rurality, lack of transportation, and financial barriers [59, 60].Some of these disparities more pronounced in remote and rural Australia, where Aboriginal and Torres Strait Islander communities are further marginalised by distance and poverty [61, 62]. Enablers of chronic disease care, such as outreach services, transportation, and referrals networks to other allied health and community groups are more likely to be effective where holistic approaches are adopted. This is consistent with data from the Australian Institute of Health and Welfare highlighting the experiences of social inequity in Aboriginal and Torres Strait Islander communities and the positive impact on health outcomes when inequities are reduced [63]. Previous studies [13, 64] have identified various obstacles to accessing primary health care services which include inadequate infrastructure, inflexible and inadequate funding to care for people holistically. Evidence also suggests that for Aboriginal and Torres Strait Islander people, travel to access health care means being separated from their country, family and social network that directly impacts their health and wellbeing as described by Milroy’s Dance of Life [65]. While government subsidies are in place, travel and accommodation costs incurred by Aboriginal and Torres Strait Islander people to access healthcare, may require upfront payment or indirect costs, perpetuating financial barriers for Aboriginal and Torres Strait Islander people living in rural and remote areas [66]. Therefore, it is crucial that primary health care initiatives take a holistic and system thinking approach to program design, considering the impact of social and cultural determinants on the health of individual, family members and their communities, with every attempt to reduce systemic barriers to access to healthcare where possible.This is only possible when funding mechanisms and models of care are flexible enough to account for local and individual contexts.

The profound impact of workforce was clear throughout this review. Recruiting, and retaining staff and effective training, were found to be key barriers to implementing and maintaining holistic patient-centred chronic disease prevention and management programs [13, 67]. Evidence has shown that Aboriginal and Torres Strait Islander people prefer support delivered by Aboriginal and/or Torres Strait Islander staff and clinicians who have a better understanding of Indigenous wellbeing [64, 68]. Despite the growth of the Aboriginal and/or Torres Strait Islander health workforce over time, this expansion has not matched the Aboriginal and Torres Strait Islander population growth [69] and increasing incidence of chronic disease. Unsurprisingly, being members of the community, they serve, Aboriginal and/or Torres Strait Islander Health Workers play an essential and unique role in delivering culturally safe and holistic care. However, a demanding work environment, low salary, inadequate support, [70] and demanding cultural brokerage with non-Indigenous colleagues [70, 71] contribute to burnout that contributes to poor retention rates of Aboriginal and Torres Strait Islander primary care staff. This requires urgent attention, by individual primary care providers and through the National Aboriginal and Torres Strait Islander Health Workforce Strategic Framework and Implementation Plan 2021 – 2031 [72]. Given the ongoing need for the non-Indigenous workforce in fulfilling workforce gaps required to deliver services for Aboriginal and Torres Strait Islander people, building cultural competence, continuing appropriate training and education pathways and strategies, providing job security and adequate remuneration are also crucial to address primary care workforce issues including the overburdening of the Aboriginal and Torres Strait Islander workforce [58, 71, 73]. Our findings highlight the need to develop the overall chronic disease workforce, with a specific focus for recruitment and retention of Aboriginal and Torres Strait Islander Health Workers and Practitioners and providing cultural safety training for all non-Indigenous staff. Alongside this, mechanisms for recognising the value and load of cultural mentorship/education should be developed. This reflects a recent research findings that showed 39% of Aboriginal and/or Torres Strait Islander workers (*n* = 1033) across Australia experienced high cultural load in terms of extra work demands and their engagement in educating others [74].

Chronic disease management requires multidisciplinary team input for effective care delivery [75]. When optimally resourced, primary care can serve a coordinating role in patient care, and effectively ensure patients have access to all allied health and specialist care they need [76]. Therefore, in order to engage Aboriginal and Torres Strait Islander people with chronic disease care and maintain continuity of care, there needs to be established, streamlined, and practitioner and patient friendly systems in place [76, 77]. Existing evidence also documented inadequate number of general practitioners and lack of specialists in rural and remote settings of Australia compared to urban or city areas which hinders individuals, particularly Aboriginal and Torres Strait Islander people, to receive timely treatment for their co-occurring conditions in an integrated care approach [78, 79]. A lack of integrated IT systems, poor infrastructure, and poor communication between primary care team members were found in this review to impede provision of such care. It is evident that strategies like GP care plans and tertiary care follow up are important sources of information for primary care providers, hospitals and patients which are supported by IT infrastructure [80]. Previous research highlighted the feasibility of system integration through utilising continuous quality improvement processes and community co-design [81]. Infrastructure investment such as internet access, in-house IT support and automated systems for follow-up care, is urgently required to ensure that patients who do present or engage with primary care in regional or remote settings, are retained in the system to enable coordinated access to the multidisciplinary care required for chronic disease management. Moreover, rapidly evolving technology such as tele-health, videoconferencing and Point-of-Care Testing may can facilitate access to the Aboriginal and Torres Strait Islander people in remote and rural areas. However, implementing these tools should be part of broader strategy rather than a substitute for solving problems faced by PHC such as workforce retention, undersupply or maldistribution issues [82]. Therefore, when implementing an integrated team care program [83] or any other integrated model of care, both barriers and facilitators identified herein should be applied to improve the continuity of care with considering the context.

Effective engagement of Aboriginal and Torres Strait Islander communities and their leadership in program design, delivery and evaluation of chronic disease programs is integral to improving Aboriginal and Torres Strait Islander health and increasing access to primary care services [84, 85]. Previous studies have identified the following factors that enable engagement with Aboriginal and Torres Strait Islander communities: employment of local Aboriginal health workforce; trust and relationships; Aboriginal and Torres Strait Islander leadership; availability of flexible services to address holistic needs of local communities; benefits of engagement in service design and delivery; cost of participation and recognition of local Aboriginal knowledge and cultural traditions on study design implementation and dissemination [84, 86–88]. These enablers align with those reported in health service research that engaged with Indigenous and marginalised communities in an international context [89]. Similarly, this review identified a range of impeding factors to engagement of Aboriginal and Torres Strait Islander people with primary health care. Key factors included: a fear or lack of trust on mainstream health facilities, lack of respect from health care providers, experiences of interpersonal and structural racism, lack of understanding of cultural differences to initiate an open discussion and a narrow concept of health that fails to consider the Aboriginal definition of health which is more comprehensive than the Western biomedical perspective of health that focuses on treating health conditions [90–92]. Evidence also shows that Aboriginal and Torres Strait Islander people in rural and remote communities do not have equitable access to PHC services, including lack of local available services to meet their holistic needs, inadequate infrastructure, high costs, long travel distance and insufficient workforce [59, 93]. Therefore, trustful, and culturally safe engagement of Aboriginal and Torres Strait Islander communities through all aspects of the program design, implementation and evaluation is essential to program success, and where possible, the Aboriginal Health Workforce and ACCHOs should be utilised.

**Policy and practice recommendations:** This study identified several policy and practice recommendations (Table 3) that need to be considered for the implementation of chronic disease prevention and management programs for Aboriginal and Torres Strait Islander people in primary care. Our recommendations align with Australia’s Primary Health Care 10 Year Plan 2022–2032 ($632.8 million new investment) that has identified three streams of work: future focused health care; person-centred primary health care supported by funding reform; and integrated care, locally delivered [94].

**Table 3:** Policy and practice recommendations

Policy level recommendations	Practice level recommendations
i. Ensure cultural competency is a core element in staff recruitment, professional development review, and other education/training opportunities	i Ensure core funding is flexible enough to be used for local priorities, including welcoming environments, local language resources or innovative models of care delivery.
ii. Recognise the role of families, carers, and communities and ensure they are included in models of care delivery	ii Support multi-disciplinary team-based care within funding models by recognising the distinct role of Indigenous and non-Indigenous health professionals and practitioners
iii. Support team-based models of care which may include Aboriginal and Torres Strait Islander Health workers/practitioners, nurses, general practitioners, midwives and allied health providers	iii. To create welcoming and supportive clinic environments, potentially including tea/coffee, hairdressing, or other services
iv. Support staff training in holistic needs assessment for chronic disease care, including social and cultural determinants of health	iv. Support strategic use of clinical information systems
v. Ensure Aboriginal and Torres Strait Islander people are able to make their own health decisions and are supported by the chronic disease team	v. Resource Communities of Practice to share best practices and mitigate burnout
vi. Support flexibility in consultation times and formats to foster genuine healthcare relationships	vi. Review capacity for MBS rebates to recognise and remunerate flexible service delivery, including outreach and extended hour services
vii. Ensure mechanisms are in place to facilitate feedback and address provider behaviours and attitudes	vii. Resource MyMedicare as a mechanism to fund high quality chronic disease care
viii. Prioritise employment of Aboriginal and Torres Strait Islander people and provide ongoing support for Aboriginal and Torres Strait Islander staff who service communities	viii. Resource ‘Deadly Choices’ incentive program for increasing quality health check uptakes by Aboriginal and Torres Strait Islander communities
ix. Develop workflows which support implementation of the National Guide to Preventative Healthcare for Aboriginal and Torres Strait Islander people and other standards of care	ix. Mechanisms for recognising the value and load of cultural mentorship/education should be developed
x. Offer flexible models of service delivery, including outreach, extended opening hours and walk-in services wherever possible	x.Provide transport services for service users to attend primary care in settings where physical access is a barrier to care
xi. Provide adequate flexible findings required to enable a place-based partnership model to deliver holistic care via making reforms through recruitment and retaining enough staff. This will ensure that people will receive the right culturally friendly care at the right time in the right setting	xi.Support the capacity of PHC drivers to have a role in health promotion and community engagement

### Strengths and limitations

Strengths of the present study include (i) the generation of an evidence summary required to guide policy and practice is a short time frame, (ii) the application of iterative process from the design to completion of review with engagement of Aboriginal and Torres Strait Islander researchers and *Thiitu Tharrmay Aboriginal and Torres Strait Islander Reference Group* members, (iii) a quality appraisal of the included studies using Aboriginal and Torres Strait Islander Quality Appraisal Tool that privileges Aboriginal and Torres Strait Islander people’s ways of knowing, being, doing and (iv) interpretation of findings validated by Aboriginal and Torres Strait Islander knowledge champions.

One limitation of this review was that search was restricted to only two databases as the decision makers seek the evidence is a short period of time and were based on peer reviewed articles published in English language. We also acknowledge that the findings might not be comprehensive as the review was conducted in short timeframe, limitations in key words used and subjected to publication bias, as we omitted published program reports, grey literature, and policy guidelines from our inclusion criteria. Moreover, our search was limited to specific databases and terms, which could result in overlooking articles present in other databases or identified through alternate search terms. Despite these limitations, this review is Aboriginal and Torres Strait Islander researchers led that allowed Indigenous perspectives and knowledge to be integrated in the evidence synthesis; ensuring findings are meaningful for the broader sector.

## Conclusion

This rapid review synthesises the barriers and enablers to designing and implementing chronic disease prevention and management programs for Aboriginal and Torres Strait Islander communities. While there is no one-size-fits-all approach to the heterogeneous Aboriginal and Torres Strait Islander communities, several policy and practice recommendations are broadly applicable to service providers. These include addressing social and cultural determinants of health, developing the Aboriginal and Torres Strait Islander and non-Indigenous chronic disease workforce, supporting multidisciplinary teams through strengthening clinical care pathways, and engaging Aboriginal and Torres Strait Islander communities in design and delivery of chronic disease prevention and management programs. This requires funding mechanisms and models of care that are flexible enough to account for local and individual context through policy and practice reforms. Moreover, enabling place-based partnerships to develop local and population-based strategies that align with community priories and aspiration is crucial for tackling increasing burden of chronic disease.

### Supplementary Information

**Additional file 1. **Study characteristics.

**Additional file 2. **Meta aggregated study findings.

## Data Availability

All available materials are available as Additional material.

## Abbreviations

ACCHOs: Aboriginal Community Controlled Health Organisations
MBS: Medicare Benefits Schedule
CVD: Cardiovascular Disease
CKD: Chronic Kidney Disease
PRISMA-P: Preferred Reporting Items for Systematic Review and Meta-analysis Protocols
STARR: SelecTing Approaches for Rapid Reviews
