# Correction: A rapid review to inform the policy and practice for the implementation of chronic disease prevention and management programs for Aboriginal and Torres Strait Islander people in primary care

**DOI:** 10.1186/s12961-024-01140-8

**Published:** 2024-04-26

**Authors:** Uday Narayan Yadav, Jasmine Meredith Davis, Keziah Bennett-Brook, Julieann Coombes, Rosemary Wyber, Odette Pearson

**Affiliations:** 1 National Centre for Aboriginal and Torres Strait Islander Wellbeing Research, Australian National University, Canberra, ACT Australia; 2 Centre for Primary Health Care and Equity, University of New South Wales, Sydney, Australia; 3 Melbourne Medical School, The University of Melbourne, Melbourne, Australia; 4 The George Institute for Global Health, Sydney, Australia; 5 Telethon Kids Institute, Perth, WA Australia; 6 South Australian Health and Medical Research Institute, Adelaide, SA Australia; 7 Faculty of Health and Medical Science, University of Adelaide, Adelaide, SA Australia

**Correction: Health Research Policy and Systems (2024) 22: 34** 10.1186/s12961-024-01121-x

Following publication of the original article it was reported that there was a typographical error due to a typesetting mistake [1]. In several instances throughout the article the phrase “Thiitu Tharrmay Aboriginal and Torres Strait Islander Reference Group members rather than Thiitu Tharrmay Aboriginal Reference Group members” was mistakenly inserted.

The original article has been updated to correct these instances to “Thiitu Tharrmay Aboriginal and Torres Strait Islander Reference Group members.”
