# Supplementary material for: A rapid review to inform the policy and practice for the implementation of chronic disease prevention and management programs for Aboriginal and Torres Strait Islander people in primary care
Source: Health Res Policy Syst. 2024 Mar 21;22:34. doi: 10.1186/s12961-024-01121-x (PMC10956197; doi:10.1186/s12961-024-01121-x)
Supplement: Supplementary file 1 — Additional file 1. Study characteristics. [file 12961_2024_1121_MOESM1_ESM.docx]

| **S.no** | **Author /year of publication** | **Research objective** | **Study design** | **Study period** | **Jurisdictions** | **Service delivery model (ACCHO / government AMS / private GP / other)** |
| --- | --- | --- | --- | --- | --- | --- |
| 1 | Cuesta-Briand et.al/2014 | To explore the impact of Indigenous status and socioeconomic disadvantage on the experience of diabetes care in the primary health setting. | Qualitative study | 1 year | WA | Aboriginal community-controlled health organisation |
| 2 | Deshmukh et.al/2014 | To study the perspectives of Aboriginal health professionals (nurses and AHWs) regarding cardiovascular risk and heart health, including their understanding of cardiovascular assessment | Qualitative study | NA | NSW | Aboriginal Medical Service Western Sydney (AMSWS) and Western Sydney Local Health District |
| 3 | Stoneman et al/2014 | Aim to compare service delivery and outcome measures between DAHS and other ACCHSs in the region and to identify strategies for improving diabetes care and the CQI process. | Mixed method | 1 year | WA | Aboriginal Health Service (benchmark) and ACCHs |
| 4 | Govil et.al/2014 | To investigate the implementation of comprehensive PHC in Australia, with a focus on the role of group work in achieving the goals of comprehensive PHC. | Mixed method evaluation | 1 year | WA | AMs |
| 5 | Bailie et.al/2015 | To examine how a national multicomponent program aimed at improving the prevention and management of chronic disease among Australian Indigenous people addressed various dimensions of access | Mixed method | 4 years | Urban, regional and remote areas of Australia. | Indigenous Health Services and the private general practice sector |
| 6 | Barrett et.al/2015 | The evaluate the effectiveness of a nurse practitioner-led approach to chronic kidney disease management in an | Implementation study | NA | NSW | Aboriginal Community Controlled Health Service |
| 7 | Reeve et.al/2015 | To describe the reorientation of a remote primary health-care service, in the Kimberley region of Australia, its impact on access to services and the factors instrumental in bringing about change. | Implementation research (mixed method) | 6 years | WA | ACCHOs, Kimberley region of Australia, |
| 8 | Bailie et.al/2016 | To describe stakeholders’ perceptions of the barriers and enablers to addressing gaps in Australian Aboriginal and Torres Strait Islander chronic illness care and child health, and to identify key drivers for improvement. | Implementation approach | NA | Australia | Australian Indigenous PHC settings |
| **9** | Askew et.al/2016 | To determine the feasibility, acceptability and appropriateness of a case management approach to chronic disease care integrated within an urban Aboriginal and Torres Strait Islander primary health care service. | Implementation Research (Mixed method exploratory) | 6 months | Southern Queensland | Queensland Government general practice |
| 10 | Schmidt et.al/2016 | To understand the experiences of engaging with the model from the perspective of the IHWs, health team members, and clients. | Process evaluation of RCT | 18 months | QLD | Apunipima Cape York Health Council+ Queensland government managed AMS |
| 11 | Conway et.al/2017 | To explore IHWs’ perceptions of the effectiveness and appropriateness of the Flinders Closing the Gap Program, as an evidence-based example of self-management support, and to explore the barriers and facilitators that IHWs experience in their workplace and communities in providing self-management support. | Case study methodology | 1 year | Five States in Australia | Aboriginal Medical Services |
| 12 | Campbell et.al/2017 | To understand enablers and barriers influencing postpartum screening for type 2 diabetes following gestational diabetes in Australian Indigenous women and how screening might be improved. | Qualitative study | 1 year | QLD | Wuchopperen Aboriginal Health Service, Cairns, and Apunipima Cape York Health Council. |
| 13 | Davy et.al/2017 | Aimed at developing a Framework to support the quality of care and quality of life of, as well as treatment for, Aboriginal and Torres Strait Islander peoples living with chronic disease. | Participatory Action Approach | 1 year | Across Australia | Aboriginal Health Services |
| 14 | Kirkham et.al/2017 | A process evaluation to report on health professional’s perceptions of models of care and related quality improvement activities since the implementation of the Partnership. | Evaluation study | 1 year | NT | Range of services including Aboriginal Health Services of NT |
| 15 | Spurling et al/2017 | Aimed to identify the priority health issues of the Inala Aboriginal and Torres Strait Islander community, and which of these might be translated into research questions answerable using computerised HA data. | Qualitative study | 6 months | QLD | Government managed AMS |
| 16 | Wood et al/2017 | Aimed to identify the processes used in systems assessment and the strengths and weaknesses of the systems in place to support the provision of quality client care using quantitative and qualitative SAT data from five consistently high- improving Indigenous PHC services. | Mixed method | 9 years | NT | Government managed AMS and one ACCHO |
| 17 | Webster et al/2017 | Aimed to identify the model of care currently experienced by Aboriginal patients as it was assumed that improvement to the model of care was the key to Aboriginal people learning to understand and manage their type 2 diabetes. | Grounded theory and participatory qualitative research | NA | NSW | AMS |
| 18 | Bailie et.al /2017 | To use clinical audit data to create a framework of the priority evidence-practice gaps, strategies to address them, and drivers to support these strategies in the delivery of recommended preventive practice. | Implementation research | NA | Across Australia | A large number of Indigenous health services |
| 19 | Canuto et.al/2018 | To identify the perceived motivators, barriers and enablers of Aboriginal and Torres Strait Islander men’s utilization of primary health care services, explore their experiences and obtain suggestions from them as to how services could be modified to improve utilization. | Indigenist Research Methods (Qualitative) | NA | SA and QLD | Mixed PHC services (AMS, ACCHO and Private GPs) |
| 20 | Kirkham et.al/2019 | To capture professionals’ perspectives on antenatal and post-partum diabetes screening and management, including enablers and barriers to care. | Qualitative study | 1 year | NT | Range of services of NT |
| 21 | Macniven et.al/2019 | To examine the feasibility and acceptability of an ECG attached to a mobile phone(IECG) screening device for atrial fibrillation in Aboriginal Controlled Community Health services (ACCHS) and other community settings. | Mixed method evaluation | 1 year | NSW, WA, and NT | ACCHS |
| 22 | Seear et al/2019 | Aim to report on the process of piloting community-led diabetes prevention program and its acceptability and feasibility through the Derby Aboriginal Health Service (DAHS). | Feasibility pilot study | 3 months | WA | Derby Aboriginal Health Service |
| 23 | Sebastian et al/2020 | Aims to examine the organisational factors of participating Indigenous primary health care (PHC) services that impacted on B.strong's uptake and implementation of those services. | Qualitative - semi-structured interviews | 3 years | QLD | - ACCHOs and government-operated health services |
| 24 | Seear et al/2020 | To discover what type of prevention program would be suitable for young Aboriginal people in and around Derby; utilise community knowledge and previous research evidence to design a preliminary lifestyle modification program consistent with community preferences; and refine the program after testing in a small exploratory pilot. | Implementation Research (Qualitative design) | 1 year | WA | Derby Aboriginal Health Service |
| 25 | Blignault et.al/2021 | Evaluation of an innovative model that leverages mainstream and Aboriginal health resources to enable safe, supported transfer of care for Aboriginal adults with chronic conditions leaving the hospital. | Qualitative study | 2 years | NSW | Government services (SWSLHD, NSW Ministry of  Health and Western Sydney University) |
| 26 | Wood et al/2021 | Aimed to explore Aboriginal Australian women’s and health providers’ preferences for a program to prevent and improve diabetes after pregnancy. | Qualitative: Semi-structured in-depth interviews | 6 months | NT | NT Government Primary Care Clinics |
