# Supplementary material for: A rapid review to inform the policy and practice for the implementation of chronic disease prevention and management programs for Aboriginal and Torres Strait Islander people in primary care
Source: Health Res Policy Syst. 2024 Mar 21;22:34. doi: 10.1186/s12961-024-01121-x (PMC10956197; doi:10.1186/s12961-024-01121-x)
Supplement: Supplementary file 2 — Additional file 2. Meta aggregated study findings. [file 12961_2024_1121_MOESM2_ESM.docx]

**Supplementary file.2. Meta aggregated study findings**

| **Meta-aggregated themes** | **Study findings** | **Supporting evidence** |
| --- | --- | --- |
| **Primary health care service attributes– enablers** | Systems for holistic support such as social referrals: Connecting to walking or art groups, housing, exercise groups and home care services (e.g., help with housework, meals or shopping) | Askew, Blignault, Webster, Conway, Seear 2019-2020 |
|  | Partnership with well-known research institute assisted in credibility of the program | Sebastian |
|  | Adequate resources or flexiblefunding required for structural changes at PHC or to meet local priorities and needs, Adequate physical space, Establishment of the Aboriginal equipment loan pool | Askew, Canuto, Reev, Davy, Bailie 2017, Blignault |
|  | Community engagement through community events, strong connections with Aboriginal and Torres Strait Islander communities, and strong Indigenous leadership | Askew, Bailie 2015, Canuto, Deshmukh, Bailie 2016, Barrett, Reev, Davy, Blignault, Bailie 2017, Sebastian, Seear 2019, Seear 2020, Sporling , Stoneman, Webster, Wood 2017, Wood 2021 |
|  | Partnership with various stakeholders (PHNs, primary care, community etc) | Kirkhan 2014 |
| **Primary health care service attributes– barriers** | Community barriers: stress at home, prioritising cultural/family needs over health | Cambell |
|  | Environmental influences on dietary health – access of unhealthy food, high availability of unhealthy food, expensive foods | Seear 2020, Wood 2021 |
|  | Lack of knowledge of what services are available | Webster |
|  | Inflexibility of the healthcare system | Wood 2017 |
|  | Housing insecurity / overcrowding | Wood 2021 |
|  | Difficult to access those patients that truly need it most | Baillie 2015 |
|  | Competing priorities of research and healthcare service delivery | Askew, Bailie 2016, Bailie 2017 |
|  | Lack of support structures for programs including leadership, funding | Conway, Bailie 2016, Bailie 2017, Schmidt, Kirkham |
|  | Lack of community input to governance/planning/program development/feeling tokenistic | Wood 2017 |
| **Chronic disease workforce enablers** | Chronic disease training and development for the primary health care workforce | Askew, Bailie 2015, Deshmukh, Cambell |
|  | Recruitment of local Indigenous Health Workers, clarification of their roles and their Involvement in Decision making +gardening relationships with community members | Askew, Deshmukh, Barrett, Davy, Bailie 2017,Schmidt, Macniven, Stoneman, Webster |
|  | Dedicated chronic disease staff with delegation of clear responsibilities and a positive team culture | Askew, Reev, Davy, Bailie 2017,Schmidt, Kirkham 2017, Stoneman, Wood 2017 |
|  | Staff felt supported, motivated, and had a shared sense of purpose to address the complex needs of patients | Askew, Sebastian |
|  | Effective recruitment and retention of staff | Bailie 2015, Bailie 2016,Bailie 2017, Wood 2017 |
|  | Cultural awareness and safety training for staff to deliver people-centred care | Davy, Sebastian, Cuesta-Briand, Cambell, Schmidt |
|  | Good supervision of trainees and corridor conversations enable two-way learning, empowerment and support | Schmidt, Askew, Barrett |
|  | Trust in local Indigenous staff and young Aboriginal facilitators as role models | Govil, Macniven, Seear 2019, Seear 2020 |
| **Chronic disease workforce barriers** | Lack of Indigenous staff/burnout/turnover of Indigenous staff | Conway, Deshmukh, Bailie 2016, Cambell, Bailie 2017, Schmidt, Kirkham 2017, Stoneman, Wood 2017 |
|  | Aboriginal Healthcare Workers feeling undervalued | Canuto, Deshmukh, Stoneman |
|  | Lack of training for non-Indigenous staff | Conway 2017, Canuto, Wood 2017, Bailie 2016, Schmidt, Govil |
|  | General staff shortages/turnover leading to lack of continuity of care | Conway, Cuesta-Briand, Deshmukh, Bailie 2016, Bailie 2017, Kirkham 2017, Stoneman, Govil, Wood 2017 |
|  | Lack of time/resources for staff to reach those who need it most | Bailie 2015, Wood 2017, Wood 2021 |
|  | Unclear role delineation | Kirkham 2019, Stoneman, Wood 2017 |
| **Patient/provider partnership enablers** | Maintaining confidentiality | Askew |
|  | shortages of specialists | Bailie 2016, Sebastian, Stoneman, Wood 2017 |
|  | Health care providers at Aboriginal services practice considering holistic needs (social and emotional support) rather than just physical health | Askew, Canuto, Cambell, Davy |
|  | Empowerment of clients through interactive learning and friendly conversations, focusing on strength-based approaches | Askew, Conway, Conuto, Deshmukh, Barrett, Cambell, Blignault, Govil |
|  | Developing trust through a collaborative approach | Conway, Macniven |
|  | Information on new services by health care providers | Bailie 2015 |
|  | More consultative time at Indigenous services (45 mins) required to maintain relationship with clients | Conuto, Davy |
|  | Client engagement strategy: Involvement of family members and carers in care | Askew, Canuto, Schmidt, Wood 2017 |
|  | Patient-centred model of care in place (Patient-led initiatives like lifestyle preventive initiatives and management plan, non-judgement communication) | Askew, Govil, Wood 2017 |
| **Patient/provider partnership barriers** | Competing priorities | Conway |
|  | Poor relationship/connection with clinician | Cuesta-Briand, Canuto, Cambel |
|  | Patients having a poor understanding of their condition | Bailie 2015, Wood 2017 |
|  | Patient discomfort with non-Indigenous services, including experiences of racism and discrimination | Canuto |
|  | Patients feeling as though the doctor cannot help with their true problems, feels superficial | Spurling |
|  | Poor patient engagement/lack understanding of the importance of their disease management | Cuesta-Briand, Kirkham 2019 |
| **Clinical care pathways enablers** | Organisational capacity in improving patient referral, coordination, and follow-up care | Askew, Bailie 2015, Canuto, Cambell, Blignault, Baile 2017, Stoneman, Wood 2017 |
|  | Staff competent to use practice software and review all progress notes made by the GPs in addition to updating participants’ medical records and attaching participants’ care plans themselves to ensure continuity of care | Askew, Stoneman |
|  | Partnership enabled service integration across the health service organisations, including a shared electronic health record system, providing disease registers, multidisciplinary care plans and a patient recall system | Reev, Askew |
|  | Telehealth by specialists (including dieticians) and e-health of opportunistic screening of CVD | Kirkhan2017, Macniven |
|  | Multidisciplinary team to address holistic needs | Davy, Blignault, Kirkham 2017, Barrett |
|  | Clinical audits improve service delivery | Bailie 2015-2017, Wood 2017, Wood 2021 |
|  | Training of staff and investment in systems development for the effective use of clinical information systems | Baile 2017, Kirkhan 2017, Bailie2016 |
|  | Educational package for GPs and other clinic staff to improve compliance with guidelines and best practices | Barrett |
|  | Flexibility in models of care that cater to the needs of women who have had GDM | Cambell |
| **Clinical care pathways barriers** | Lack of integrated systems for recalls and referrals e.g. multiple IT platforms, mix of paper and electronic records | Conway, Kirkham 2017, Stoneman 2014, Wood 2017 |
|  | Poor infrastructure e.g. internet access | Bailie 2016, Schmidt, Macniven |
|  | Staff shortages e.g. lack of staff to implement new pathway systems, staff not trained adequately | Bailie 2016, Sebastian, Stoneman, Wood 2017 |
|  | Lack of communication between hospitals and primary care | Govil |
|  | Inconsistency of model of care | Webster |
|  | Poor links between health centres and communities including IT systems, relationships | Bailie 2016, Schmidt, Kirkham 2017, Kirkham 2019 |
| **Accessibility to primary health care services enablers** | Point of reference for clients who needed assistance to seek other services | Askew |
|  | Flexible system of seeing GPs/nurses without a prior appointment | Cuesta-Briand, Wood 2021 |
|  | Transportation support | Bailie 2015, Bailie 2017, Blignault |
|  | Previous family incidents (demise of family members because of conditions) motivated to seek preventive health services, or referrals supported by family members | Canuto, Conway |
|  | Incentives for health checks e.g. motivational texts | Canuto, Seear 2019, |
|  | Reduced waiting times | Stoneman |
|  | 24 hr-culturally safe services, flexible primary care services (Service and staff proximity to home and ability to provide family support) both within and outside of health care facilities. | Canuto, Davy, Govil |
|  | Providing services at no cost (medications, nicotine patches, etc) | Cuesta-Briand, Bailie 2015 |
|  | Provision of multiple services(specialised clinics, supplementary services and preventive activities, including glucose monitors) at no cost | Bailie 2015, Govil, Kirkham 2017 |
|  | Outreach services (saving clients time and cost) [ medication delivery, general visits to build trust and opportunistic screening ] | Askew, Cuesta-Briand, Bailie 2015, Wood 2021 |
|  | Community education (including nutritional resources for pregnancy) and financial support, along with education for behaviour change in a supportive family environment | Askew, Barrett, Davy, Govil, Kirkham 2017 |
| **Accessibility to primary health care services barriers** | Geographical inaccessibility – lack of transport both to access health professionals, and to make healthy lifestyle choices | Askew, Conway, Cuesta-Briand, Bailie 2015, Canuto, Cambell, Schmidt, Govil, Webster, Wood 2017, |
|  | Staff inaccessibility – lack of appointments, long waiting periods, lack of leadership, staff turnover | Cuesta-Briand, Canuto, Kirkham 2019, Sebastian, Wood 2021 |
|  | Information inaccessibility – unaware of what services exist, poor promotion of services | Cuesta-Briand, Bailie 2015, Deshmukh |
|  | Financial inaccessibility – services expensive, lack of funding, unable to afford medications | Cuesta-Briand, Canuto, Sebastian, Webster, Bailie 2015 |
|  | Patient inaccessibility – e.g. patient not having a phone, fixed address | Bailie 2015, Govil |
|  | Psychological inaccessibility – fear, denial, lack of motivation, shame, putting others needs first | Cambell |
|  | Technological inaccessibility – no access to computer, unreliable internet access, old computers | Sebastian |
|  | Physical inaccessibility – wheelchair access, disability access, visual disability, lack of rooms/space in clinics, | Wood 2017, Stoneman, Webster |
|  | Recognition of the need to offer broader social determinants of health support but lack of resourcing to do so | Ballie 2015, Cambell |
| **Culturally acceptable and safe services enablers** | Provision of culturally safe services and culturally appropriate education materials (e.,g. story booklet, Indigenous designed graphics/images/voices), an increase number of client friendly services for patients were recognised as patient-related facilitators | Askew, Conway, Davy, Webster, Wood 2017, Wood 2021 |
|  | Culturally appropriate care for Aboriginal and Torres Strait Islander peoples, which contributed to the development of a mutually respectful relationship systems approach to address racism, experience of colonisation, improving culturally safe environment etc | Askew, Webster |
|  | Gender-specific strategies for delivered services, such as separate exercise groups ,health assessment days +private consultation areas for males and females | Bailie 2015, Seear 2019, Wood 2017, Wood 2021 |
|  | Employed both male and female staff to ensure gender sensitivity | Bailie 2015 |
|  | Indigenous people hired as outreach workers played crucial role of cultural brokage that made services more acceptable and assisted with access to care | Bailie 2015, Macniven |
|  | Staff responding to, and understanding family, community, cultural and spiritual responsibilities and obligations | Davy, Spurling, Webster |
|  | Indigenous co-design, feedback from community, incorporation of community thoughts in program development | Blignault, Sebastian, Webster, Wood 2017, Wood 2021 |
| **Culturally acceptable and safe services barriers** | Physical inaccessibility – inappropriate space for care e.g. living arrangement not suitable for home care | Askew |
|  | Fear of discrimination/racism – avoid mainstream services, previous negative experiences | Conway, Cuesta-Briand, Bailie 2015, Canuto, Webster |
|  | Fear of diagnosis | Conway |
|  | Poor self-management due to traumatic past / mental illness | Conway |
|  | Feeling that services are rushed/business focused rather than holistic - particularly health assessments | Bailie 2015, Spurling |
|  | Long wait times/competing priorities for time e.g. family, work | Canuto, Govil |
|  | Lack of Indigenous specific services | Canuto, Webster, Wood 2017 |
|  | Language barrier | Canuto 2018 |
|  | Culturally inappropriate services | Canuto, Cambell |
|  | Heterogeneity of Indigenous populations | Canuto, Cambell, Bailie 2017, |
|  | Poor health literacy/understanding of condition/importance of appointments | Schmidt |
|  | Lack of space or availability for gendered privacy | Stoneman, Webster, Wood 2017 |
